# MicroRNA-338-5p alleviates neuronal apoptosis via directly targeting BCL2L11 in APP/PS1 mice

**DOI:** 10.18632/aging.104005

**Published:** 2020-10-21

**Authors:** Junhua Li, Danhua Li, Huatao Zhou, Guiyun Wu, Zhijie He, Wenhua Liao, Yujuan Li, Yaowei Zhi

**Affiliations:** 1 Department of Anesthesiology, Sun Yat-sen Memorial Hospital, Sun Yat-sen University, Guangzhou 510120, China; 2 Department of Pediatrics, Sun Yat-sen Memorial Hospital, Sun Yat-sen University, Guangzhou 510120, China; 3 Department of Cardiology, Sun Yat-sen Memorial Hospital, Sun Yat-sen University, Guangzhou 510120, China; 4 Department of Intensive Care Unit, Sun Yat-sen Memorial Hospital, Sun Yat-sen University, Guangzhou 510120, China; 5 Laboratory of RNA and Major Diseases of Brain and Hearts, Sun Yat-sen University, Guangzhou 510120, China; 6 Guangdong Province Key Laboratory of Brain Function and Disease, Zhongshan School of Medicine, Sun Yat-sen University, Guangzhou 510080, China

**Keywords:** Alzheimer's disease, MicroRNA-338-5p, neuronal apoptosis, BCL2L11

## Abstract

MicroRNAs have become pivotal modulators in the pathogenesis of Alzheimer’s disease. MiR-338-5p is associated with neuronal differentiation and neurogenesis, and expressed aberrantly in patients with cognitive dysfunction. However, its role and potential mechanism involved in Alzheimer’s disease remain to be elucidated. Herein, we showed that the expression of miR-338-5p decreased in APP/PS1 mice, accompanied by the elevation in the expression level of amyloid β, which indicated a reverse relationship between Alzheimer’s disease progression and miR-338-5p. In addition, lentiviral overexpression of miR-338-5p through intrahippocampal injection mitigated the amyloid plaque deposition and cognitive dysfunction in APP/PS1 mice, suggesting a protecting role of miR-338-5p against the development of Alzheimer’s disease. Moreover, miR-338-5p decelerated apoptotic loss of neurons in APP/PS1 mice. MiR-338-5p decreased neuronal apoptosis *in vitro* induced by amyloid β accumulation, which was attributed to the negative regulation of BCL2L11 by miR-338-5p, since the restoration of BCL2L11 eliminated the protective role of miR-338-5p against neuronal apoptosis. Taken together, all of these results may indicate miR-338-5p as an innovative modulator in the pathogenesis of Alzheimer’s disease, and also suggest that the protective effect of miR-338-5p on neuronal apoptosis may underlie its beneficial effect on APP/PS1 mice.

## INTRODUCTION

Alzheimer's disease (AD), the most common neurodegenerative disease and the leading cause of dementia in elderly people, is characterized by hyperphosphorylated aggregated tau protein as neurofibrillary tangles, amyloid β (Aβ) protein deposition as senile plaque and neurodegeneration. At present, the treatment for AD is extremely expensive, and no effective treatment for AD has been developed since the etiology of AD is still undetermined. To develop effective treatments to decelerate progression of AD or to prevent development of the disease, a new molecular target is imperatively needed.

Slow and progressive degeneration of neurons in brain regions associated with learning and memory ability leads to personality changes, damage to normal social and emotional behaviors, and cognitive decline [1–3]. The apoptotic loss of neurons underlying memory impairments is considered as an early pathological hallmark of AD and is believed to be caused by accumulating Aβ peptides [4, 5]. Progressive degeneration of neurons was found to promote Alzheimer-like neurodegeneration in transgenic APP/PS1 mic [6], indicating that protecting against apoptotic loss of neurons may have therapeutic benefit in retarding the progression of AD.

MicroRNAs (miRNAs) are small noncoding RNAs, 18 to 22 nucleotides long, involved in the post-transcriptional control of gene expression by binding to the 3'UTR of the target gene mRNA to promote degradation or inhibited translation of mRNAs [7]. A single miRNA can interact with multiple downstream mRNAs because their binding sequences are relatively small [8]. Therefore, miRNAs represent a critical group of gene network modulators. Many miRNAs are found to be specifically expressed in the brain. Particularly, several miRNAs have been shown to modulate neurological development, including neuronal migration [9], neurogenesis [10], axon and dendrite development [11, 12]. Even mild abnormal expression in miRNA expression may impair the brain function as shown by the previous studies [13, 14]. Consequently, finding out the relationship between miRNAs and AD provides a new insight in studying the neuropathology and pathogenesis of AD. Indeed, accumulating evidence has shown the great potential of several miRNAs as biomarkers in AD diagnosis [15, 16]. Moreover, miRNAs are associated with AD pathology mechanistically via distinct mechanisms, such as synaptic damage, Tau pathology and the modulation of Aβ level [15, 17]. Despite these studies, further investigation is still required to explore the potential contribution of miRNAs to the pathogenesis of AD.

Recently, miR-338-5p has been shown to engage in regulating neuronal placement and polarity [18] and neuronal outgrowth [19]. Moreover, increased level of miR-338-5p enhances the neuronal repair after spinal cord injury [20] and decreased expression of miR-338-5p contributes to the development of AD [21]. In the present study, we demonstrated an protective effect of miR-338-5p on cognitive dysfunction through utilizing transgenic APP/PS1 mice, which may associate with the decelerated apoptotic loss of neuron via decreasing neuronal apoptosis. We provide the evidence that miR-338-5p may function as a negative modulator in the progression of AD, proposing a promising innovative strategy for the therapeutic intervention of AD.

## RESULTS

### The expression of miR-338-5p significantly decreased in APP/PS1 mice during the progression of AD

**Figure 1:**
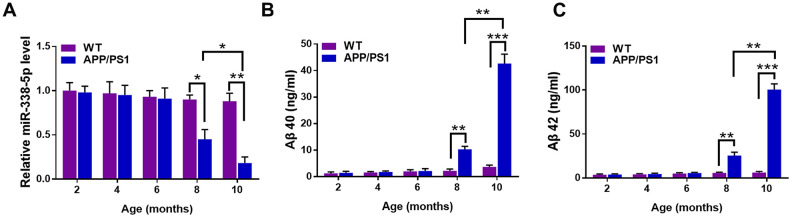
**The expression of miR-338-5p decreased in APP/PS1 mice during AD progression.** (**A**) qRT-PCR analysis of miR-338-5p expression in the brains of wild-type (WT) and APP/PS1 mice with increasing age. Data are presented as relative to that of 2-month-old mice. (**B**, **C**) ELISA analysis of Aβ 40 (**B**) and Aβ 42 (**C**) level in the brains of WT and APP/PS1 mice with increasing age. Data are presented as ng Aβ 40 or Aβ 42 per ml total protein samples. Results are presented as mean ± SD. n = 6 in each group. *P < 0.05; **P < 0.01; ***P<0.001.

To elucidate the correlation between AD pathology and miR-338-5p, we first detected the expression pattern in the brain of APP/PS1 mice at the age of 2,4,6,8 and 10 months old [22]. The results of qRT-PCR suggested that the expression of miR-338-5p declined dramatically in 8-month-old APP/PS1 mice, which became further lower in 10-month-old APP/PS1 mice, in comparison to the wild-type (WT) mice (Figure 1A). However, among the brains of APP/PS1 mice aged 2, 4 and 6 months old, no significant change was observed in miR-338-5p expression level (Figure 1A). Furthermore, the expression level of Aβ 40 (Figure 1B) and Aβ 42 (Figure 1C) also increased significantly in 8-month-old APP/PS1 mice, which were further shown remarkable elevation in 10-month-old APP/PS1 mice, compared with wild-type mice. All of these synchronous changes between miR-338-5p and Aβ production in APP/PS1 mice indicated the level of miR-338-5p may be negatively correlated with the pathogenesis of AD.

### MiR-338-5p attenuated cognitive dysfunction in APP/PS1 mice

**Figure 2:**
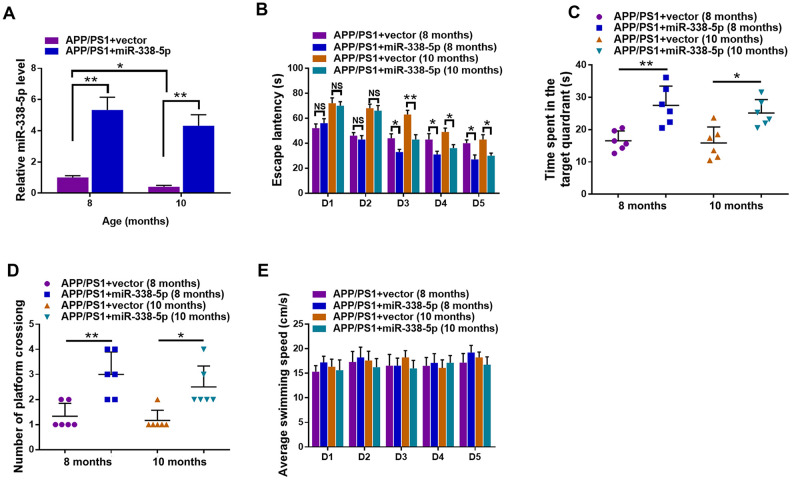
**Lentiviral overexpression of miR-338-5p through intrahippocampal injection improved cognitive dysfunction in APP/PS1 mice.** (**A**) qRT-PCR analysis of miR-338-5p expression in the brain. Data are presented as relative to that of 8-month-old mice infected with lentiviral empty vector. (**B**) Spatial learning of 8-month-old or 10-month-old APP/PS1 mice was detected as escape latency at different days after training in water maze. (**C**, **D**) Spatial memory of 8-month-old or 10-month-old APP/PS1 mice was assessed by probe trials 24 h after the last training session. The number of platform crossing (**C**), time spent in target quadrant (**D**) and swimming speed (**E**) of 8-month-old or 10-month-old APP/PS1 mice were recorded. Results are presented as mean ± SD. n = 6 in each group. *P < 0.05; **P < 0.01; ***P<0.001.

We reversed the downregulation of miR-338-5p in the brain of APP/PS1 mice aged 8-month-old and 10-month-old through intrahippocampal injection of lentiviral vector overexpressing miR-338-5p to test whether miR-338-5p affects cognitive deficits in APP/PS1 mice [23]. The effectiveness of lentiviral vector overexpressing miR-338-5p in the brain of APP/PS1 mice was validated by qRT-PCR (Figure 2A). By performing the Morris water maze, we then evaluated whether miR-338-5p affects the ability of spatial learning and memory in APP/PS1 mice. In MWM, the relative average escape latency of five training days and the escape latency of each training day were used to show the learning ability. Significant differences were observed in escape latency from day 3 onwards between group APP/PS1+vector and group APP/PS1+miR-338-5p (Figure 2B). Furthermore, in the probe trail, the number of platform-crossing and target quadrant traveling time indicated the memory activities. Moreover, compared to the control group, the time spent in the target quadrant (Figure 2C) and frequency to cross the platform (Figure 2D) were significantly increased in group APP/PS1+miR-338-5p indicated by the probe trials 24 h after the last training session. It is noteworthy that there is no significant difference in the average swimming speedamong the groups (Figure 2E), suggesting that miR-338-5p-ameliorated behavioral performances of APP/PS1 mice resulted from cognitive processes, instead of non-cognitive behavioral activities. Taken together, the results of MWM tests demonstrated that overexpression of miR-338-5p in the brain attenuated impaired spatial learning and memory in APP/PS1 mice.

### MiR-338-5p attenuated the amyloid plaque deposition of APP/PS1 mice

**Figure 3:**
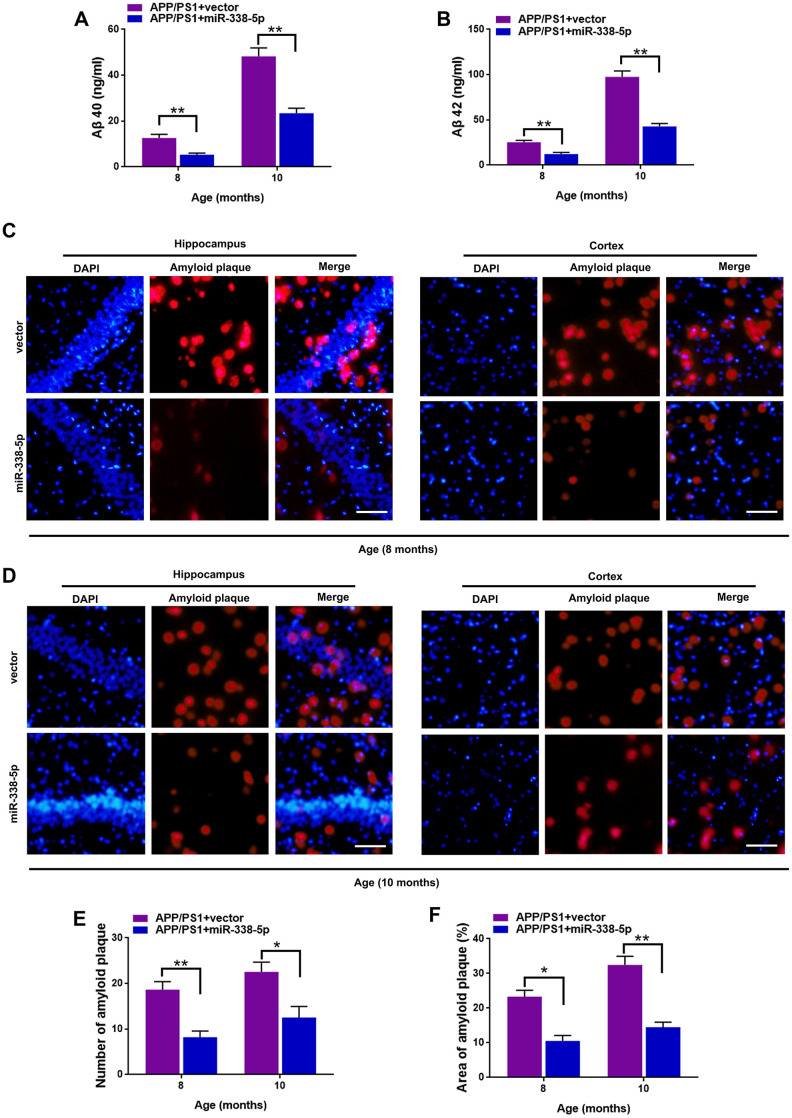
**MiR-338-5p attenuated amyloid plaque deposition in APP/PS1 mice.** (**A**, **B**) ELISA analysis of Aβ 40 (**A**) and Aβ 42 (**B**) level in APP/PS1 mice. Data are presented as ng Aβ 40 or Aβ 42 per ml total protein samples. (**C**, **F**) Thioflavin-S was used to stain the brain sections to show the number and area of amyloid plaques in hippocampus and cortex. Representative images of plaques in hippocampus and cortex aged 8-month-old (**C**) or 10-month-old (**D**). (**E**, **F**) Quantification analysis of the number (**E**) and area (**F**) of amyloid plaque. The plaques were shown with red fluorescence and cell nuclei were stained with blue fluorescence by DAPI. Scale bar=50 μm. Results are presented as mean ± SD. n = 6 in each group. *P < 0.05; **P<0.01.

We determined whether miR-338-5p decreased amyloid plaque deposition, a hallmark pathologic change scattered in AD brain, to further explore the association between miR-338-5p and AD progression [24]. Compared with control group, the expression levels of Aβ 40 (Figure 3A) and Aβ 42 (Figure 3B) tested by ELISA assay showed remarkable reduction in APP/PS1 mice when overexpressing miR-338-5p in the brain. To further validate these results, Thioflavin-S staining was performed to detect the amyloid plaque in brain slices. Consistent with the results of ELISA assay, both the number (Figure 3C–3E) and area (Figure 3C, 3D, 3F) of amyloid plaque deposition in hippocampus and cortex of APP/PS1 mice significantly decreased when overexpressing miR-338-5p. These results suggested that miR-338-5p decreased amyloid plaque deposition in APP/PS1 mice, which was consistent with its protective effect on cognitive deficits in APP/PS1 mice (Figure 2).

### MiR-338-5p retarded the apoptotic loss of neurons in APP/PS1 mice

**Figure 4:**
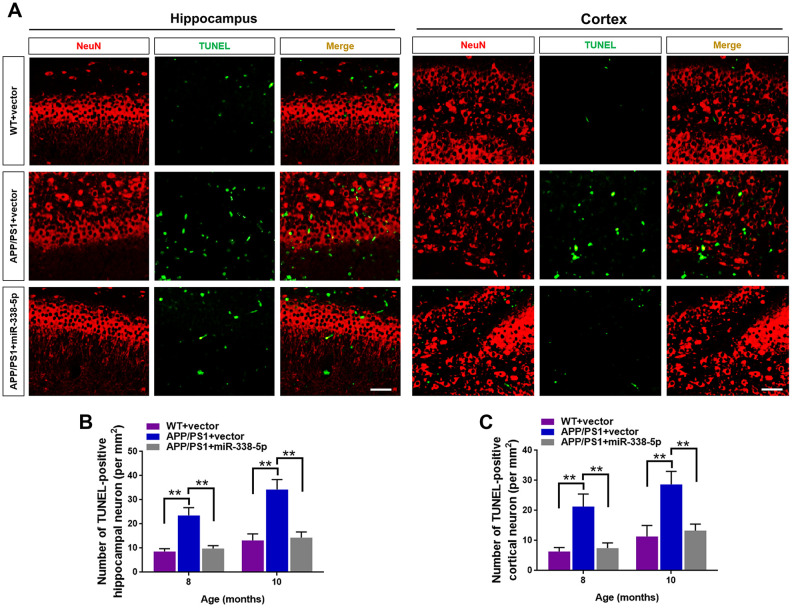
**MiR-338-5p retarded apoptotic loss of neurons in APP/PS1 mice.** (**A**) The representative immunofluorescent images of TUNEL-positive neurons in 8-month-old WT and APP/PS1 mice. Green staining indicated TUNEL-positive cells and red staining indicated neurons. (**B, C**) Quantification of TUNEL-positive neurons in hippocampus (**B**) and cortex (**C**) of 8-month-old or 10-month-old WT and APP/PS1 mice. Scale bar=50 μm. Results are presented as mean ± SD. n = 5 in each group. **P < 0.01.

Several miRNAs show the potential to attenuate the apoptotic loss of neurons, such as miR-23a and miR-96 [25, 26]. Furthermore, neuronal loss and the ensuing cognitive dysfunction are inevitable consequences, which are also vital contributing factors to AD progression [27]. As shown by the TUNEL assay, the number of apoptotic neuron in the hippocampus and cortex significantly increased in 8-month-old and 10- month-old APP/PS1 mice, in comparison to wild-type mice (Figure 4A–4C). Interestingly, we showed that the increased number of apoptotic neurons in the brain of APP/PS1 mice was remarkably recovered by overexpressing miR-338-5p (Figure 4A–4C). Hence, these results suggested that miR-338-5p decelerated the apoptotic loss of neurons in APP/PS1 mice.

### MiR-338-5p ameliorated neuron apoptosis induced by Aβ accumulation

**Figure 5:**
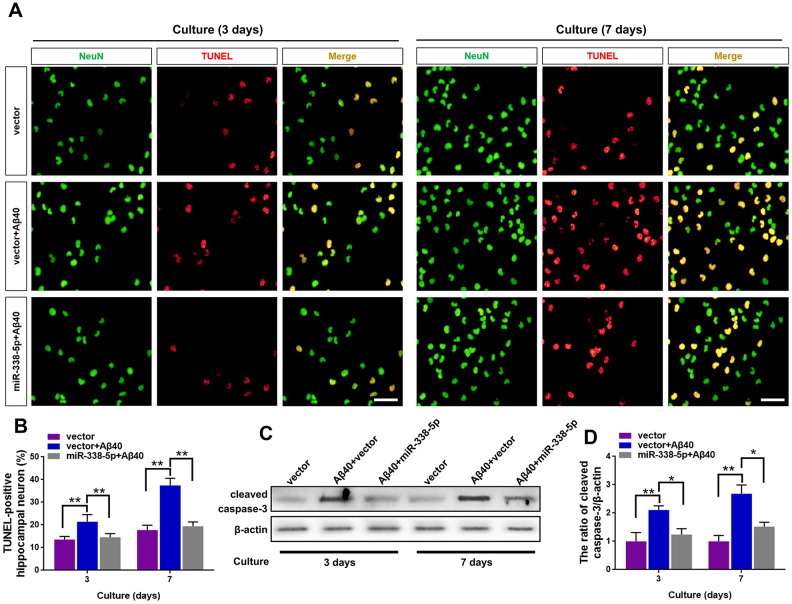
**MiR-338-5p rescued neuron apoptosis induced by Aβ accumulation.** (**A**–**C**) Primary hippocampal neurons were isolated infected with lentiviral miR-338-5p expressing vector or lentiviral empty vector. Two days later, neurons were cultured for consecutive 3 or 7 days with or without 5 mM Aβ40. (**A**, **B**) The representative immunofluorescent images (**A**) and quantification (**B**) of TUNEL-positive hippocampal neurons *in vitro*. (**C**, **D**) The representative western blot images (**C**) and quantification analysis of cleaved caspase-3 expression (**D**). Scale bar=50 μm. Results are presented as mean ± SD. n = 3 in each group. *P < 0.05; **P<0.01.

We isolated the primary hippocampal neurons from APP/PS1 mice brain, culturing them *in vitro* and then treated them with Aβ40, which can cause cell death of neurons by prolonged accumulation [28] to further probe into the ameliorating role of miR-338-5p in neuron. As shown by the TUNEL assay, the increased number of apoptotic neurons treated by Aβ40 treatment for consecutive 3 and 7 days was remarkably mitigated when overexpressing miR-338-5p in neurons (Figure 5A, 5B). To further validate these results, we then detected the expression level of cleaved caspase-3. Indicated by the results of Western blot, Aβ40-induced increased cleaved caspase-3 expression in neurons was rescued by overexpressing miR-338-5p (Figure 5C. 5D), which was consistent with the results of TUNEL assay. Therefore, miR-338-5p protected against neuron apoptosis caused by Aβ40 accumulation, indicated by these *in vitro*o experiments.

### MiR-338-5p attenuated neuron apoptosis by directly targeting BCL2L11

**Figure 6:**
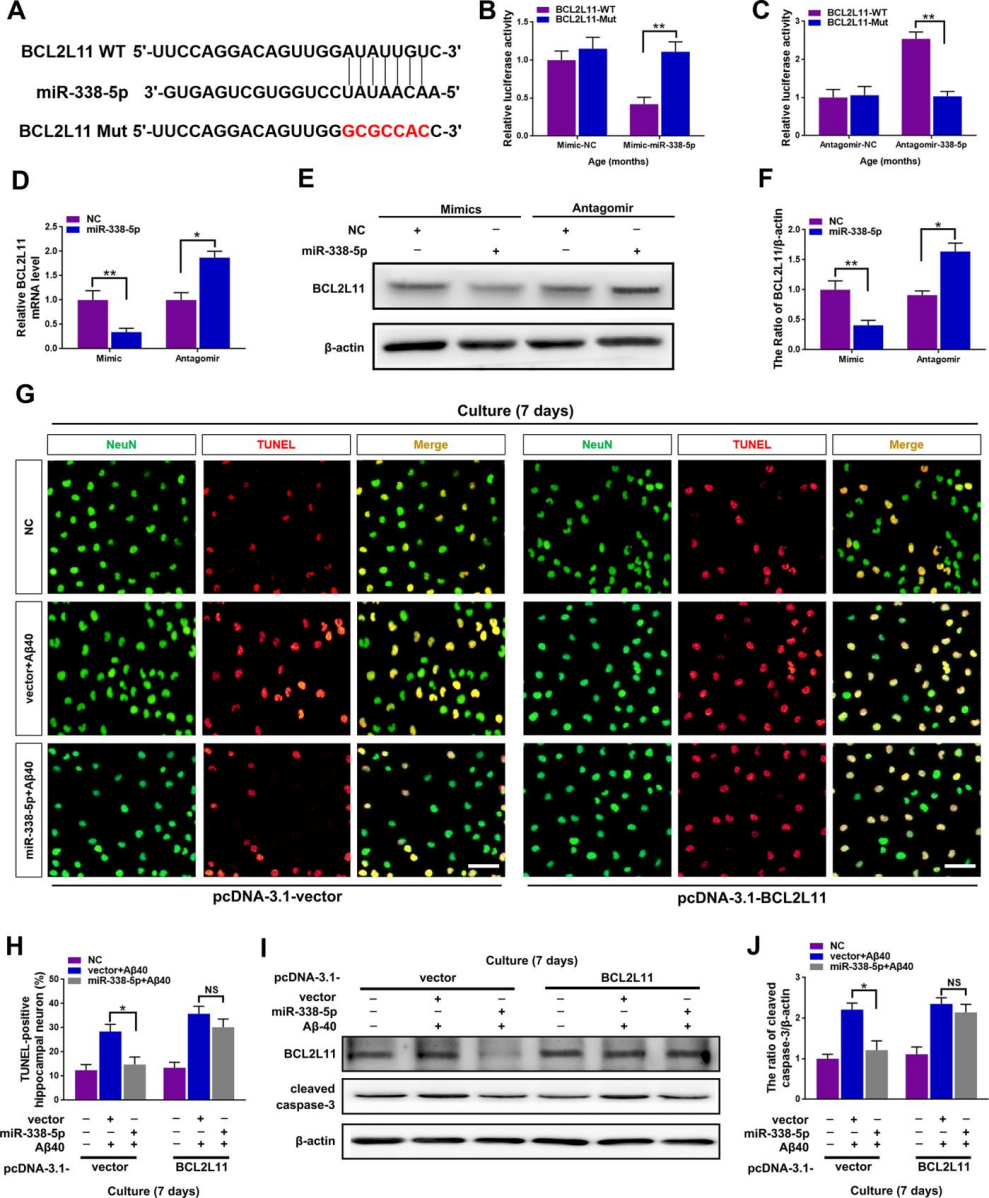
**miR-338-5p ameliorated neuronal apoptosis by directly targeting BCL2L11.** (**A**) TargetScan was used to predict the binding sites of miR-338-5p within the 3ʹ-UTR of BCL2L11. (**B**) Overexpressing miR-338-5p resulted in a remarkable decrease in luciferase activity of BCL2L11-WT and exerted no effect on luciferase activity of BCL2L11-Mut in primary hippocampal neurons. (**C**) Silencing miR-338-5p caused a significant increase in luciferase activity of BCL2L11-WT and exerted no effect on luciferase activity of BCL2L11-Mut in primary hippocampal neurons. (**D**–**F**) Relative BCL2L11 mRNA level (**D**) and protein expression (**E**, **F**) of primary hippocampal neurons transfected with NC mimic or miR-338-5p mimic, or NC antagomir or miR-338-5p antagomir determined by qRT-PCR and Western blot respectively. (**G**–**I**) Primary hippocampal neurons were transfected with pcDNA3.1-vector or pcDNA3.1-BCL2L11. Two days later, neurons were cultured for consecutive 7 days with or without 5 mM Aβ40. (**G**, **H**) The representative immunofluorescent images (**G**) and quantification (**H**) of TUNEL-positive hippocampal neurons *in vitro*. (**I**, **J**) The quantification of TUNEL-positive hippocampal neuron *in vitro*. The representative western blot images (**I**) and quantification analysis (**J**) of cleaved caspase-3 expression. Scale bar=50 μm. Results are presented as mean ± SD. n = 3 in each group. *P < 0.05; **P<0.01.

To further clarify the underlying mechanism of miR-338-5p decelerating neuron apoptosis under Aβ40 treatment, Targetscan was used to conduct targeting prediction analysis based on bioinformatics [29]. We focused on the BCL2-like 11 (BCL2L11) (Figure 6A), since it has been previously reported to contribute to neuron apoptosis [30], The 3ʹ-UTR of BCL2L11 with wild-type or mutant seed sequence recognition sites was cloned into a luciferase reporter to determine whether BCL2L11 was a direct target of miR-338-5p. The results showed that the overexpressing miR-338-5p resulted in a significant reduction in the luciferase activity of the plasmid carrying BCL2L11 3ʹUTR-WT, while luciferase activity in cells transfected with the BCL2L11 3ʹUTR-Mut plasmid did not change significantly (Figure 6B). Conversely, silencing miR-338-5p showed remarkable increase in luciferase activity of wild-type 3’UTR of BCL2L11 with the mutant construct unaffected (Figure 6C). These results suggest that BCL2L11 can be targeted by miR-338-5p directly. Furthermore, we next determined whether miR-338-5p inhibits BCL2L11 expression in neurons. The results showed that overexpressing miR-338-5p reduced BCL2L11 expression, and conversely, silencing miR-338-5p increased BCL2L11 expression in neurons, further supporting its negative role in modulating BCL2L11 expression (Figure 6D–6F). Consistent with the *in vitro*o experiments, overexpressing miR-338-5p also significantly decreased the expression level of BCL2L11 in the brain of APP/PS1 mice (Supplementary Figure 1). We then hypothesized the suppressed BCL2L11 expression contributed to the ameliorating role of miR-338-5p against Aβ40-induced neuron apoptosis. To verify this hypothesis, BCL2L11 expression in neurons was restored through overexpression mediated by transient transfection. Astonishingly, we found that the protecting effect of retarding apoptotic loss of neuron (Figure 6G, 6H) and ameliorating neuron apoptosis elicited by miR-338-5p (Figure 6I, 6J) were completed reversed along with BCL2L11 restoration. Therefore, all of these results suggested that the anti-apoptotic effect of miR-338-5p on neurons treated by Aβ40 depended on the expression of its target, BCL2L11, unveiling the important role of miR-338-5p/BCL2L11 axis in attenuating neuron apoptosis treated by Aβ40 *in vitro*.

## DISCUSSION

AD is one of the most common neurodegenerative diseases and accounts for 80% of dementia cases in people aged older than 65 years [31]. The disease is clinically characterized by dementia, loss of cognitive functions and massive neurodegeneration. Although in the past 20 years, great progress has been made in understanding the pathogenesis of AD and great efforts have been made in developing drug therapy, current treatments, such as N-methyl d-aspartate receptor antagonist and acetylcholinesterase inhibitors, fail to exert satisfactory curative effect [32]. The changed expression profile of miRNAs in patients with AD and its association with the pathology of AD have been postulated for many years, including the revealed significant role of some miRNAs in modulating synaptic plasticity, Tau and β-amyloid peptide toxicity [33–35]. Thus, miRNAs can be potential therapeutic targets and promising biomarkers for AD treatment. In addition, AD is characterized by various pathological changes in central nervous system, including the apoptotic loss of neurons [36]. Recently, increasing attention has been paid to the role of neuron apoptosis in the pathogenesis of AD [37]. In the current study, we uncovered an alleviating effect of miR-338 on AD pathology, and may correlate with its protective role in retarding apoptotic loss of neurons during AD progression. The evidence supporting the above postulated finding is described as follows: First, the expression of miR-338-5p decreased in APP/PS1 mice, which synchronized with the Aβ 40 and Aβ 42 accumulation. Moreover, through intrahippocampal injection of lentivirus, miR-338-5p overexpression attenuated impaired spatial learning and memory in APP/PS1 mice. Furthermore, miR-338-5p mitigated amyloid plaque deposition retarded apoptotic loss of neurons in APP/PS1 mice. Finally, miR-338-5p protected against neuron apoptosis *in vitro* induced by Aβ treatment.

In the present study, miR-338-5p expression showed significant reduction in the brain of APP/PS1 mice aged 8-month-old and 10-month-old, which synchronous with the remarkable increase of Aβ 40 and Aβ 42. This result indicates that the decreased expression of miR-338-5p is related to AD progression. Intriguingly, miR-338-5p expression is significantly decreased in the plasma exosome of elder people [38]. Although no obvious decline was observed in the expression of miR-338-5p in 10-month-old WT mice, the decline of miR-338-5p in APP/PS1 mice was accelerated, and the underlying mechanisms remains to be elucidated. The reason may be that the decreased expression of miR-338-5p was related to neuron integrity [18]. The concentration of miR-338-5p in serum has been raised as a potential diagnostic biomarker in colorectal cancer [39] and retinoblastoma [40]. Given that it is imperative to find non-invasive biomarkers for detecting the pathogenesis of AD, exploring whether the serum miR-338-5p shows analogic tendency in AD patients would have great clinical significance.

As shown by the previous studies, selective overexpression or inhibition of miR-338 in cortical neuron improved or damaged the dendritic complexity and axon outgrowth [19]. Additionally, silencing miR-338-5p led to the loss of neuronal polarity and significantly decreased the number of neurons [18]. Nevertheless, it’s reported that the decreased expression of miR-338-3p correlates with neuronal survival [41]. Consistent with the above studies, in the present study, miR-338-5p overexpression rescued impaired spatial learning and memory and decreased amyloid plaque deposition in APP/PS1 mice, which suggested that decreased miR-338-5p expression contributed to cognitive dysfunction and Aβ accumulation in the progression of AD, and also implied that miR-338-5p might be a promising therapeutic target for AD treatment.

It has been hypothesized that apoptosis is involved in the neuron loss in AD development [42, 43]. For example, Caspase-3 immunoreactivity was activated in AD brain [42, 44] and in APP/PS1 mice [45, 46]. Intriguingly, cytochrome c released from mitochondria was caused by Aβ42 [47], which activated Caspase-3 activity and induced apoptosis, thus providing a potential mechanism for intraneuronal neuron loss induced by Aβ42. All of these studies unveiled a pivotal role of neuronal death mediated by apoptosis in neurodegenerative diseases, including AD. In this study, as shown by immunofluorescent assay, we showed that miR-338-5p over-expression was negatively associated with neuron loss in APP/PS1 mice. In any case, these results associate the decrease of apoptotic neurons with the alleviating effect of miR-338-5p on AD development in APP/PS1 mice. Meanwhile, we suppose that neuron may not be the sole cellular target of miR-338-5p in central nervous system. Further investigations are still needed to clarify whether other cell types, such as endothelial cells, microglia and astrocytes, contribute to the function of miR-338-5p in AD progression.

Under treatment of CaMKII inhibitor, BCL2L11 can induce neuronal apoptosis [48]. The inhibition of BCL2L11 also reduced neuron apoptosis [49]. Consistent with the role of BCL2L11 in promoting neuron apoptosis, we showed that miR-338-5p ameliorated neuron apoptosis by targeting BCL2L11 directly in an *in vitro* system simulating Aβ accumulation.

## CONCLUSIONS

In summary, we provide evidence that miR-338-5p may function as a promising miRNA regulator in AD development and also associate its ameliorating effect with protection against apoptotic loss of neurons, thus shedding a light on the importance of counteracting neuronal apoptosis in improving the progression of AD.

## MATERIALS AND METHODS

### Animals

In this study, all experimental protocols and procedures were approved by the Medical School of Sun Yat-Sen University, and were conducted in strict accordance with National Institutes of Health Guidelines for the use of experimental animals. Wild-type mice (male, C57BL/6) and APP/PS1 transgenic mice (male, APP/PS1) were purchased from Beijing Vital River Laboratory Animal Technology Co., Ltd. All animals were housed in polypropylene cages and the relative humidity was maintained at 50 ± 10% and the room temperature was maintained at 22°C, with a 12 h light-dark cycle. All animals were sacrificed by exposure to carbon dioxide at the end of the experiment.

### Western blot

Samples of tissues and cells were lysed in RIPA lysis buffer with phosphatase and protease inhibitors. Protein samples were subjected to 10% SDS-PAGE and transferred to PVDF membranes. 5% non-fat milk in 0.1% TBST buffer was used to blocked membranes at 4 °C overnight. The membranes were subsequently incubated with antibodies cleaved caspase-3 (Cell Signaling Technology #9661, 1:1000), BCL2L11 (Cell Signaling Technology #2933S, 1:1000), β-actin (Cell Signaling Technology #3700S, 1:2000). The protein–antibody complex was detected with HRP-conjugated secondary antibodies and enhanced chemiluminescence. Image J software (GE Healthcare, USA) was used to perform the analysis of western blot.

### Quantitative RT-PCR

Total RNA was extracted from cultured cells in accordance with the manufacturer’s protocol (Invitrogen). By using Superscript First-Strand cDNA Synthesis Kit (18080-051, Invitrogen, Carlsbad, CA), Total RNA (1 μg) was reverse transcribed into cDNAs. SYBR Premix Ex Taq II kit (DRR081A, TAKARA, Japan) on LightCycler 480 System (Roche, Switzerland) was used to performed quantitative RT-PCR.

### Intrahippocampal injection of lentivirus

APP/PS1 mice aged 7-month-old or 9-month-old were anesthetized with chloral hydrate and placed in a stereotactic frame (stereotaxic apparatus 51600, Stoelting, USA), positioned in a stereotaxic instrument. Then, 2 μL lentivirus miR-338-5p, or lenti-vector was injected into the hippocampus bilaterally using the following coordinates: −2.7 mm dorsal/ventral, −2.7 mm anterior/posterior, ±3.2 mm medial/lateral from the bregma [23]. By using a syringe (Syringe pumps 51600z, Stoelting, USA) and a 27-gauge needle, the preparation was injected at a speed of 0.5 μL/min over a period of 4 min. One month after intrahippocampal injection of lentivirus, mice were used for subsequent biochemical and behavior analyses.

### Measurement of Aβ40 and Aβ42

An enzyme-linked immunosorbent assay (ELISA) was performed to detect the concentration of Aβ40 and Aβ42 in the hippocampus of APP/PS1 mice and wild-type mice. Briefly, mice were first anesthetized by chloral hydrate and we removed the brain tissues quickly. Next, brain tissues were homogenized with RIPA buffer on ice, and then centrifuged at 12,000 rpm for 10 min to collect the supernatants. Aβ40 Mouse ELISA Kit (Invitrogen) and Aβ42 Mouse ELISA Kit (Invitrogen) were used to measure the concentration of Aβ40 and Aβ42 in strict accordance with the manufacturer's protocols.

### Behavioral test

Morris water maze (MWM) was used to assess the spatial memory performance of mice according to the previous report [50]. In brief, animals were first placed in the water maze pool (temperature 22 ± 1°C, depth 50 cm, diameter 150 cm) for 2 days to adapt to the environment. In training trials, mice were released from three different quadrants and trained to find the hidden platform for five consecutive days. The platform was removed on the sixth day, and platform-crossing times and target quadrant traveling time were recorded, which indicated the memory ability of the animals. In probe trials, the platform was removed 24 hours after the last training. Mice were set free to swim at the starting point for 60 s, and duration in the target quadrant and the number of platform crossing were recorded. ANY-maze software (Stoelting Co.) was used to track the animal behavior automatically.

### Tissue preparation and immunofluorescence

Mice were anesthetized with chloral hydrate and perfused with 4% paraformaldehyde transcardially. The brain tissues were paraffin embedded and sectioned at the thickness of 5 μm. For thioflavin-S staining, we used 0.2% thioflavin-S (T1892, Sigma-Aldrich) to stain brain sections for 10 min. After the brain sections were washed with PBS for three times, the brain sections were photoed by an IX53 fluorescence microscope (Olympus). Image J software (GE Healthcare, USA) was employed to analyze the quantification of images.

### TUNEL assay

For TUNEL staining of brain sections, the anti-NeuN antibody (Cell Signaling Technology #24307S, USA, 1:200) diluted in PBS was incubated with the sections overnight at 4°C. Goat anti-rabbit antibody 546 (red, Santa Cruz Biotechnology, USA, 1:200) were then biotinylated the tissue sections for 60 min. TUNEL staining was performed in accordance with the manufacture’s instructions of TUNEL system kit (Promega, USA). Image-pro Plus software (GE Healthcare, USA) was employed to analyze the number of neurons stained positively for TUNEL and NeuN in hippocampus and cortex by two persons blinded to the treatments.

For TUNEL staining of neuron, the cultured primary neurons were washed once with PBS, fixed with 4% paraformaldehyde at room temperature for 1 h and permeabilized using 0.1% Triton X-100. The anti-NeuN antibody (Cell Signaling Technology #24307S, USA, 1:200) was incubated with the fixed neuron overnight at 4°C. Then TUNEL reaction mixture were incubated with the fixed neurons at 37 °C for 1 h. DAPI was added to the wells for 5 min to stain nuclear after rinsing the cells with PBS. We counted the TUNEL-positive cells manually, and calculated the percentage of positive cells for each sample.

### Culture and treatment of primary hippocampal neurons

The hippocampi were removed from the brains under a light microscope. Hippocampal neurons were dissociated with DNase and 0.125% trypsin. Then the neurons were seeded in 6-well culture plates for Western blotting at a density of 1 × 10^5^ cells/cm^2^ or in poly-d-lysine-coated glass coverslips for immunocytochemistry staining at a density of 1 × 10^4^ cells/cm^2^. Cultures were maintained in neurobasal A medium (Invitrogen, Carlsbad, CA) containing 0.5 mM glutamine and 2% B27 supplement in an incubator (95% air, 5% CO2) at 37 °C. For the treatment of Aβ40 (FC3-018-01, Phoenix Pharmaceuticals), the neurons were cultured for consecutive 3 or 7 days with and without 5 mM Aβ40. Fresh medium was replaced every 2 days in the presence and absence of 5 mM Aβ40 until the end of the experiment.

### Statistical analysis

The Shapiro–Wilk test was used to test whether the data were normally distributed and Levene's test was used to confirm that the data had no significant heterogeneity of variance. Data were presented as means ± SD and analyzed by one-way ANOVA followed by Tukey’s post-hoc test. Sample size was calculated by using the SPSS 11 software to achieve an 80% power at a significance level of 0.05. The GraphPad Prism software (version 7.0, CA, USA) was used to conduct the statistical analyses. In all cases, statistical significance was accepted at *P* < 0.05.

## Supplementary Material

Supplementary Figure 1
