# Supplementary material for: MicroRNA-338-5p alleviates neuronal apoptosis via directly targeting BCL2L11 in APP/PS1 mice
Source: Aging (Albany NY). 2020 Oct 21;12(20):20728–42. doi: 10.18632/aging.104005 (PMC7655176; doi:10.18632/aging.104005)
Supplement: Supplementary Figure 1 [file aging-12-104005-s001..pdf]

SUPPLEMENTARY FIGURE

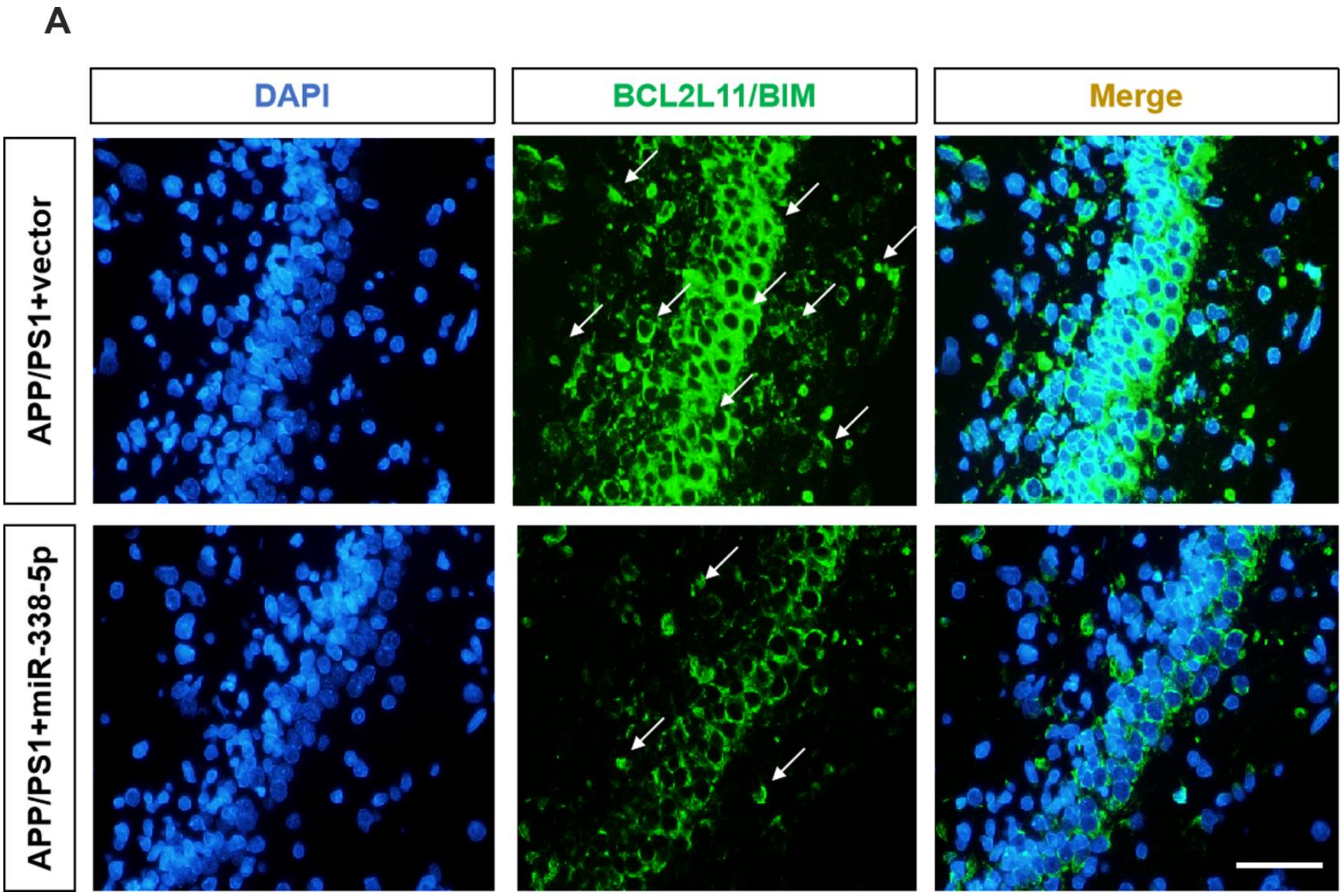

Supplementary Figure 1. The representative immunofluorescence images of BCL2L11/BIM staining in APP/PS1 mice. (**A**) Scale bar = 50µm.
